# Effects of Receiver Beamforming for Vital Sign Measurements Using FMCW Radar at Various Distances and Angles

**DOI:** 10.3390/s22186877

**Published:** 2022-09-12

**Authors:** Shahzad Ahmed, Junbyung Park, Sung Ho Cho

**Affiliations:** Department of Electronic Engineering, Hanyang University, Seoul 04763, Korea

**Keywords:** frequency modulated continuous wave radar, beamforming, vital sign extraction, digital healthcare

## Abstract

Short-range millimeter wave radar sensors provide a reliable, continuous and non-contact solution for vital sign extraction. Off-The-Shelf (OTS) radars often have a directional antenna (beam) pattern. The transmitted wave has a conical main lobe, and power of the received target echoes deteriorate as we move away from the center point of the lobe. While measuring vital signs, the human subject is often located at the center of the antenna lobe. Since beamforming can increase signal quality at the side (azimuth) angles, this paper aims to provide an experimental comparison of vital sign extraction with and without beamforming. The experimental confirmation that beamforming can decrease the error in the vital sign extraction through radar has so far not been performed by researchers. A simple, yet effective receiver beamformer was designed and a concurrent measurement with and without beamforming was made for the comparative analysis. Measurements were made at three different distances and five different arrival angles, and the preliminary results suggest that as the observation angle increases, the effectiveness of beamforming increases. At an extreme angle of 40 degrees, the beamforming showed above 20% improvement in heart rate estimation. Heart rate measurement error was reduced significantly in comparison with the breathing rate.

## 1. Introduction

Microwave radars have enabled many wireless sensing applications such as gesture recognition [1,2], human behavior monitoring [3], and vital sign recognition [4]. Amongst these applications, human vital sign monitoring has a great potential for the healthcare industry. In the modern era, in-home health monitoring has gained huge public interest since early diagnosis can avoid many medical emergency situation [5]. Radar sensors provide a continuous and non-contact solution to monitor vital signs. This non-contact and non-invasive nature of radar-based vital sign extraction can bring several applications such as cardiopulmonary diagnostics, treatment, athletes’ physical condition monitoring, elderly monitoring and search-and-rescue operations [6,7]. Radar can also provide in-home human vital sign monitoring systems for self-diagnostic purposes. Another type of non-contact sensor is a camera sensor [8,9] where video frames are analyzed to extract cardio-pulmonary movement. However, camera sensors have an associated privacy issue [10]. Users do not feel comfortable being watched all the time.

Radar sensors have brought us many medical applications other than vital sign monitoring. For instance, radar is used for activity monitoring in the elderly community [11]. In addition, the authors in [12] provided a feasibility study for sensing medical emergencies in the home environment.

The micro-movements of the human chest caused by heart beat and respiration creates a periodic vibration which can be sensed with a radar sensor. Early attempts for human vital sign extraction dates back to 1970s when Lin [13] provided an early feasibility study recording chest vibrations using radar. Today, several implementations of human vital sign extraction exist.

In most of the radar-based vital sign extraction, the human participants are often placed at the zero-degree angle-of-arrival (AoA). This is because radars have directional beam patterns, and the detection performance degrades as we move away from the zero-degree AoA., Beamforming is designed to overcome this limitation.. It aims to increase the reflections coming from the direction of the target while reducing the reflections from the other arrival angles. For a specific AoA, the phase values across a multiple input and multiple output (MIMO) radar system is adjusted in such a way that the beamformed signal collectively increases the signal-to-noise ratio (SNR).

The radar cross section (RCS) of the human body is ranged approximately between 0.5 m^2^ to 3 m^2^ [14] which is in fact less than a metal plate of 0.15 m^2^. In fact, the actual cardio-pulmonary reflections have an RCS of less than 0.5 m^2^ [15]. In such scenarios, targeting a radar beam towards human participants using an MIMO radar may increase the quality of the radar returns corresponding to chest movement. An MIMO radar consists of multiple transmitters (TX) and receivers (RX) which are collectively used to increase the overall signal quality.

## 2. Related Work

As stated earlier, while measuring vital signs, a human participant is often located at the zero AoA. For instance, the authors in [6] used frequency modulated continuous wave (FMCW) radar with a new phase-unwrapping strategy for human vital sign extraction in various sleeping positions. Similarly, Yoo et al. [16] used FMCW radar for children vital sign detection and age-group classification using convolutional neural network (CNN). Another research work compared vital sign extraction at four different chest positions [17] and concluded that a radar sensor placed at the front side of a human participant is the optimum position. A few attempts have been made to extract vital signs through walls [18] which have also enabled the use of radar for search and rescue operations [19].

Recently, a few research works have shown the initial feasibility of multiple-target vital sign extraction using the MIMO beamforming assembly [20,21,22,23]. For example, in reference [20], three humans located at three different distances were localized, and their vital signs were extracted independently. Similarly, the authors in [21] also detected multiple human vital signs with a radar sensor. However, the participants were located at different distances. Cardillo et al. [24] reviewed research related to the applications of MIMO radars for health-related applications. Their work suggests that beamforming is often used not only to extract vital signs, but also to localize human targets. In the literature, both multi-static [25] as well as mono-static [26] radars have been employed for medical applications.

In the aforementioned studies, the effect of beamforming on vital sign detection at different angles and distances is not discussed so far. Experimental confirmation and quantification of beamforming for vital sign detection is yet to be explored. In this study, we aim to perform the experimental analysis to quantify the effectiveness of beamforming on the side angles, that is to say, when the AoA is not zero. A detailed experimental setup considering different distances and angles was designed and data with and without beamforming was simultaneously collected. The new and the main contributions of this article are as follows:

- This paper is the first study presenting an experimental comparison of vital sign extraction with and without beamforming at different distances and angles. To the best of the authors’ knowledge, the effectiveness of beamforming for vital sign measurement has not been quantified by researchers so far. An experimental confirmation of the usefulness of beamforming for vial sign measurements is presented. Vital signs are measured with radar and references sensors simultaneously, and the measurement difference is quantified for with and without beamforming cases.
- We provide an experimental setup and strategy to verify the effectiveness of beamforming by considering different distances and angles to extract vital sign. In addition, a range–angle map to extract the AoA of the target is also performed. The extracted AoA can further be used to perform the beamforming operation.
- This study aims to show the practicality of computationally low-complexity beamforming algorithm to improve vital sign extraction.

The rest of the manuscript is organized as follows. Section 3 provides the materials and methods of the research work which involves an explanation regarding the experimental design used in the paper and the signal pre-processing and beamforming details. Section 4 deals with the experimentation and results based on the methodology formulated in Section 3. Finally, Section 5 and Section 6 provides the discussion and conclusion, respectively.

## 3. Materials and Methods

### 3.1. Designed Experimental Setup

**Figure 1:**
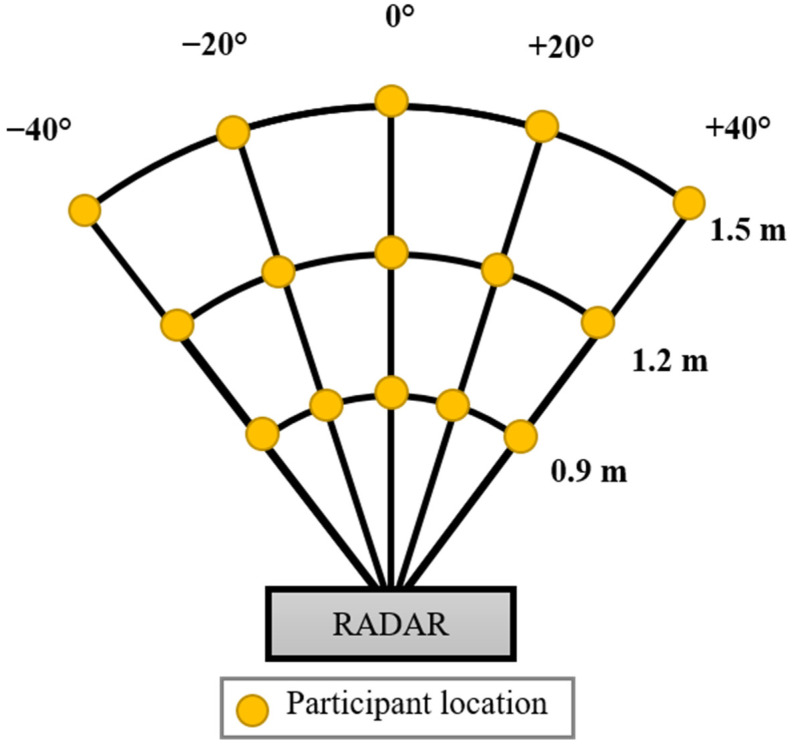
Designed experimentation to collect vital sign data at different distances and angles.

For experimental comparison, a carefully designed experimental setup is necessary for data capturing. We designed the experimental setup shown in Figure 1 where several different angles and distances are considered. The experimental setup comprised five discrete data capturing points at a distance of 0.9, 1.2 and 1.5 m each. These distances were chosen given the fact that in most of the research related to vital sign extraction, human subjects are near the radar [16,17]. Long-range vital sign measurements are often used in search and rescue operations. The five points in our experimental setup are separated by an angle of 20 degrees and range between positive and negative. Angles above 40 are beyond the scope of our experimentation. In total, there are 15 data capturing points which are highlighted in yellow in Figure 1. Note that the human participants were at rest during data capturing. Since the main objective of this research is to compare the performance of vital sign extraction in two cases that are without and with beamforming, we captured vital sign data simultaneously in both cases for the sake of a fair comparison.

### 3.2. FMCW Radar Signal Processing

**Figure 2:**
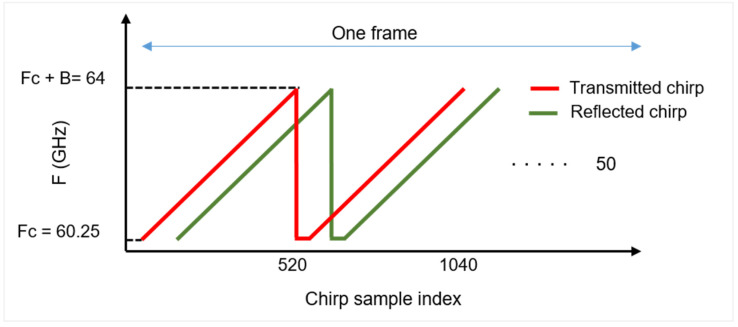
Chirp of FMCW radar spanning a bandwidth of B Hz.

FMCW radar transmits a saw-tooth modulated signal whose frequency increases linearly with time known as chirp [2]. The transmitted signal $x\left(t\right)$ can be expressed as [27,28]

(1) $$x\left(t\right)=exp\left\{j2\pi \left(f_{c}t+\frac{1}{2}\frac{B}{T}t^{2}\right)\right\},$$

where B represents the bandwidth of the chirp, T represents the time period, and $f_{c}$ represents the starting carrier frequency. In (1), the term *B/T* defines the ramp of chirp which is directly proportional to the bandwidth and inversely proportional to the time period of one chirp. In a single radar frame, there are several chirps being transmitted together as shown in Figure 2. As observed in Figure 2, there exists a delay between the transmitted chirp shown in red and the received chirp shown in green. The corresponding received signal *y*$\left(t\right)$ from a moving target will be

(2) $$y\left(t\right)=exp\left\{j2\pi \left(f_{c}\left(t-\Delta t\right)+\frac{B}{T}{\left(t-\Delta t\right)}^{2}\right)\right\},$$

where Δt represents the round trip time-delay and can be calculated based on the radial velocity $v_{r}$, distance R, and speed of light c such that

(3) $$\Delta t=2\left(\frac{R+v_{r}t}{c}\right).$$

It should be noted that the round trip delay contains the time taken by the signal to reach the target and then reach the receiving antenna.

At the receiver, the received signal is multiplied with the copy of the transmitted signal, and the high frequency values from the resulting terms are ignored to recover a low-frequency signal [29]. This circuitry is often termed as the mixer, and the output of the mixer is called the intermediate frequency (IF) signal [30].

(4) $$\mathrm{IF}\left(t\right)=exp\left\{j2\pi \left(f_{c}t+\frac{B}{T}\left(\Delta t\right)t-\frac{B}{2T}t^{2}\right)\right\}.$$

**Figure 3:**
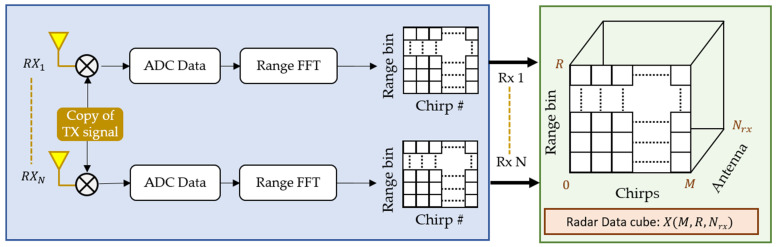
Extraction of radar data cube (RDC) from the received signal at different antennas.

For the case of an MIMO radar, the IF signal at each receiver is extracted independently. The overall signal processing chain for an MIMO radar consisting of $N_{RX}$ receiving channels is shown in Figure 3. As shown in Figure 3, the received signal at each channel expressed as *y*$\left(t\right)$ according to Equation (1) is mixed with a copy of transmitted signal to form the low frequency signal $\mathrm{IF}\left(t\right)$. The IF signal is digitized separately at each RX channel, and fast Fourier transform (FFT) is taken. Afterwards, a 2D matrix is constructed for each receiver where the peak in the FFT range bin will resolve the target (human chest) location. The signal from each RX channel is combined at the end to form a radar data cube (RDC) matrix X, whose dimensions are

(5) $$\mathrm{Dimensions}\;\left(X\right)=\left[M,R,\;N_{RX}\right],\;$$

here M,R, and $N_{RX}$ presents the number of chirps, range FFT size, and number of (virtual) RX antennas, respectively. This radar RDC matrix is generally used to extract target information in different domains such as the range–time domain, the velocity–time domain, and the range–angle domain. Next, we present the beamforming operation being used in this research.

### 3.3. Beamforming with OTS Radar

**Figure 4:**
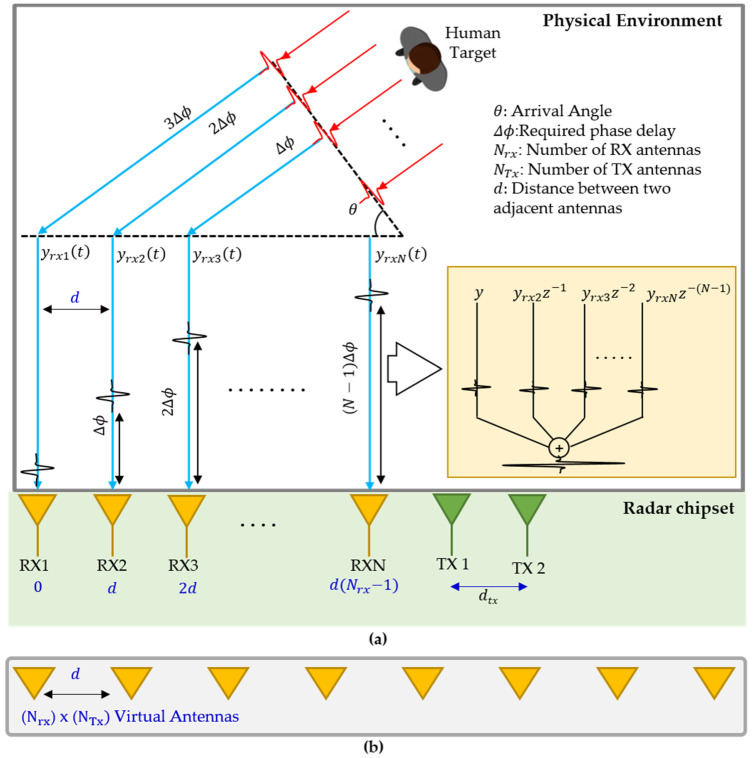
(**a**) MIMO system in consideration for vital sign extraction and (**b**) the virtual MIMO array.

Figure 4a describes the theoretical details of RX beamforming with N receiving channels. With multiple (two) transmitters placed at different locations, a distinct non-coherent signal is transmitted by each TX antenna. In order to perform RX beamforming, these transmitted signals are orthogonal in nature [26]. The RX chain receives the signal being reflected by the target (human chest). With multiple TX and RX, the number of elements of a virtual uniform linear array (VULA) will be [31]

(6) $$\mathrm{VULA}=N_{TX}N_{RX}.$$

In Equation (6), the $N_{TX}$ and $N_{RX}$ represent the number of transmitters and receivers, respectively. Figure 4b represents the virtual array corresponding to a setup shown in Figure 4a.

Since the distance between two adjacent antennas is far less that the distance between the human target and the receiver, it can be said that the reflected signals for each channel travel in parallel to each other.

In this study, we used receiver beamforming to increase the signal coming from the target direction while minimizing the other directions. As explained earlier, the MIMO radar setup for capturing vital signs is shown in Figure 4a. Assume that we have *N_rx_* receiver channels separated by a distance *d* which is often a fraction of the wavelength *λ*, and the AoA between the radar and the target is *θ*, the receiver near the target will receive the reflection first. The main objective of beamforming is to align all the received signals and sum them up as expressed in Figure 4a. A constant phase increment is required to steer the beam towards the desired reflection. The delay Δϕ(θ) for a specific Rx antenna and an angle θ can be found as

(7) $$\Delta \phi (\theta )=\left(n_{rx}-1\right)\frac{2\pi (d)\sin\left(\theta \right)}{\lambda },$$

where $n_{rx}$ represents *n*th RX antenna in the virtual array for beamforming, and d represents the distance between two RX antennas. Note that the OTS FMCW radar under consideration has two TX and four RX antennas which collectively provide 8 virtual RX antennas. As stated earlier, the distance between the two adjacent RX antennas of the OTS FMCW radar is λ/2. The above equation can be simplified as

(8) $$\Delta \phi (\theta )=\pi \left(n_{rx}-1\right)\sin\left(\theta \right).$$

**Figure 5:**
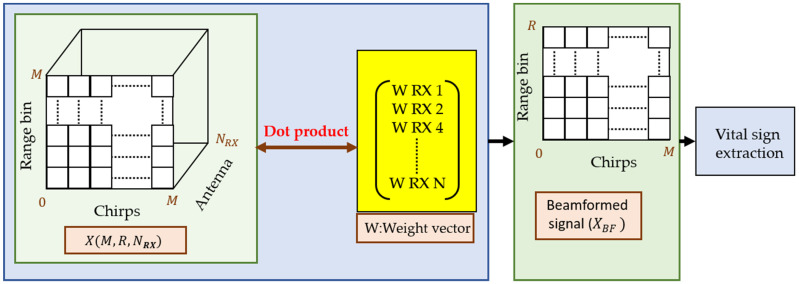
Beamforming operation: data from multiple receivers is multiplied with a weight vector to obtain a unified signal.

For a specific angle θ, the required delay term Δϕ(θ) in terms of a complex exponential is multiplied with the RDC matrix X, as expressed in Figure 2. The weight vector of all the eight RX antennas of the OTS FMCW radar under consideration will be

(9) $$W=\exp\left\{-j\pi \left(n_{rx}-1\right)\sin\left(\theta \right)\right\},$$

where W is the weight vector to be multiplied with the matrix X having dimensions equal to number of considered RX antennas. The resulting dot product of X and W can be expressed as

(10) $$X_{BF\;}=X^{H}W,$$

where H represents the Hermitian matrix. In (10), the BF represents the beamformed signal which is the combination of each individual RX channel. The process from Equations (7)–(10) is summarized in Figure 5. As explained earlier, the weight vector is calculated according to Equation (8) and then multiplied with the RDC expressed in (9). The resulting values are summed up (by taking the dot product) to form a unified signal $X_{BF}$ which increases the reflection for a specified arrival angle. This signal is then used to extract human vital signs.

The MIMO FMCW radar considered in this study is designed by Texas Instruments (TI), Texas, TX, USA (IWR6843 FMCW radar). Table 1 lists the remainder of the technical specifications of the OTS radar. As shown in Table 1, with two TX and four RX we have created a VULA of eight elements.

**Figure 6:**
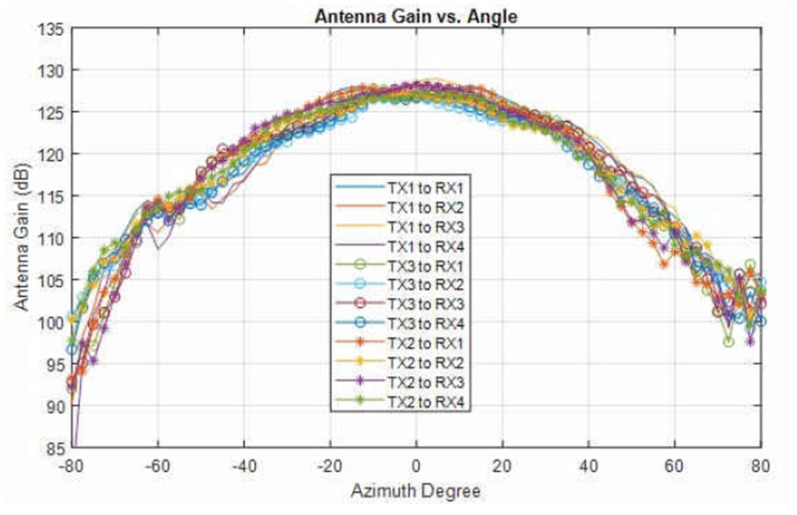
Radiation pattern for the pairs of TX and RX antennas (re-printed from [32]).

The antenna pattern of all the transmitter and receiver pairs is shown in Figure 6 [32]. The gain in terms of horizontal angle (azimuth) shown in Figure 6 suggests that as we move away from the zero-degree AoA, the antenna gain decreases significantly.

### 3.4. Range–Angle Map Extraction

**Figure 7:**
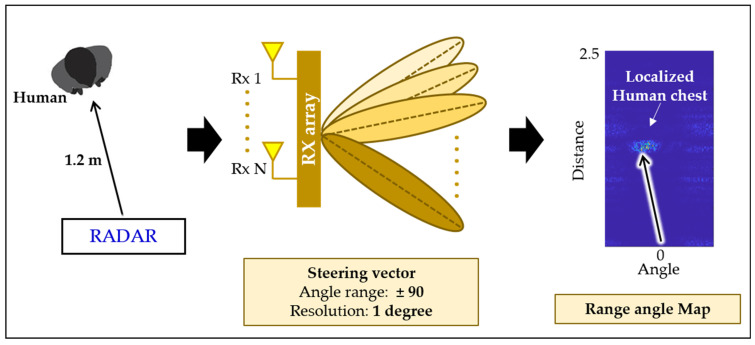
Target localization in the range–angle map using a beam steering vector.

At the receiver side, beam steering is often performed by scanning each angle separately to find out the angle-of-arrival. To validate the effectiveness of the designed beamformer, we scanned each angle between −90 and +90 degree with a step-size of 2 degrees. The resulting plot is termed as the range–angle map. The designed beam scanner and corresponding output for one of the under-consideration angles is shown in Figure 7. As shown in Figure 7, the output of the beam scanner will have a high signal concentration at 40 degrees in the range–angle map.

### 3.5. Vital Sign Extraction Algorithm

The stepwise adopted vital sign extraction is as follows:

- Step 1: Collect the IF signal corresponding to the chest reflection for each receiving channel.
- Step 2: Perform range–FFT at each channel.
- Localize the target in the range–angle map and find the angle-of-arrival.
- Step 3: Perform beamforming to combine the signals from each channel.
- Step 3: Remove clutter from the signal using a loop back iterative filter [25]. For big movements, simple mean removal tilter works well for removing clutter. However, for vital signs, a filter is often deployed since the chest movement itself is small.
- Detect the human location.
- Extract and accumulate the phase from each radar frame at the point where the human is located.
- Use two separate band-pass filters to extract breathing and heart rates.
- Use a moving mean filter to further reduce the noise in the radar recordings.

Please note that in our experiment, since we performed experiments with and without beamforming, step 3 will be excluded while extracting vital sign without using beamforming. Instead, only RX-1 is utilized, and the rest of the data is discarded in that case.

## 4. Experimentation and Results

### 4.1. Participants

In the interest of generality, we invited six participants and data were captured at each point shown in Figure 1. The average age and weight of the participants was 28 kg and 72.6 years old, respectively. The rest of the details are listed in Table 2. An informed consent form was duly signed by the participants and the principle investigator of this research. Local ethics committee at Hanyang University approved the research methodology under the Institutional Review Board (IRB) number HYU-2021-01-015.

### 4.2. Actual Experimental Setup

**Figure 8:**
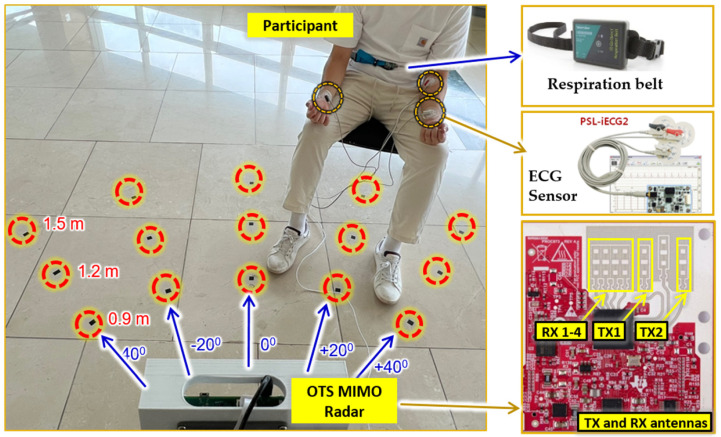
Actual experimental setup consisting of radar, ECG sensor, and respiration belt. The participant is sitting at a distance of 1.5 m from the radar with a horizontal axis of +20 degrees.

Figure 8 shows the actual experimental environment created based on the idea presented in Figure 1. The human participant was sitting on a chair wearing a respiration belt and an ECG sensor in a room at one of the specified points. The ECG sensor (PSL-iECG2) is made by PhysioLab in South Korea, whereas the respiration belt (GDX-RB) is made by Vernier in USA. The red highlighted points at different distances and angles show all the 15 data points in consideration. An experienced researcher oversaw all the data capturing to ensure that the quality of the data was adequate. Figure 8 also demonstrates the OTS radar antenna layout showing two TX and four RX antennas to create a VULA of eight elements.

### 4.3. Range–Angle Maps with Beam Steering

**Figure 9:**
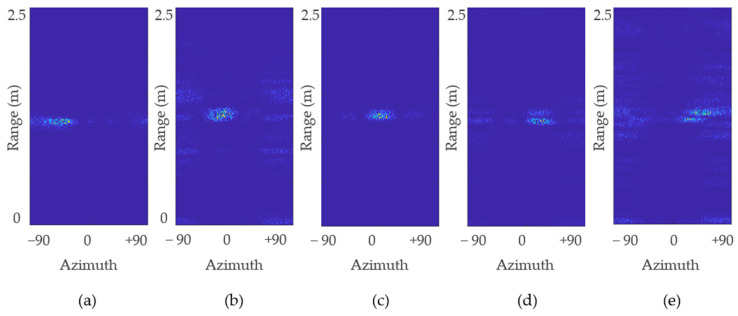
Range–angle map for: (**a**) −40°; (**b**) −20°; (**c**) 0°; (**d**) 20°; and (**e**) 40° measured when the target was located at 1.2 m.

Figure 9 shows the rrange–angle map calculated according to the above formulated beam steering methodology. The horizontal axis represents the angle ranging between ±90 degree, and vertical axis represents the distance between the human and the radar. As shown in Figure 9, the target located approximately at 1.2 m at a different angle was localized correctly by the adopted beamforming algorithm.

It can be seen in Figure 9 that the human target does not appears as a rigid dot in the rrange–angle map since the human chest is not a single point target. An average value of all the data points shown in Figure 9 provides the calculated AoA. Table 3 presents the difference between the desired angle of the target (human chest) and the angle being calculated by using the beam scanner shown in Figure 7. Note that the term desired angle represents the ideal arrival angle for each case, as shown in Figure 1. Table 3 suggests that for each case, the difference was less than 8 degrees. Since the aim of our work is to verify the effectiveness of beamforming to extract vital signs, we used the desired angle as input to the beamformer to avoid additional errors.

### 4.4. Phase Synchronization of Target

**Figure 10:**
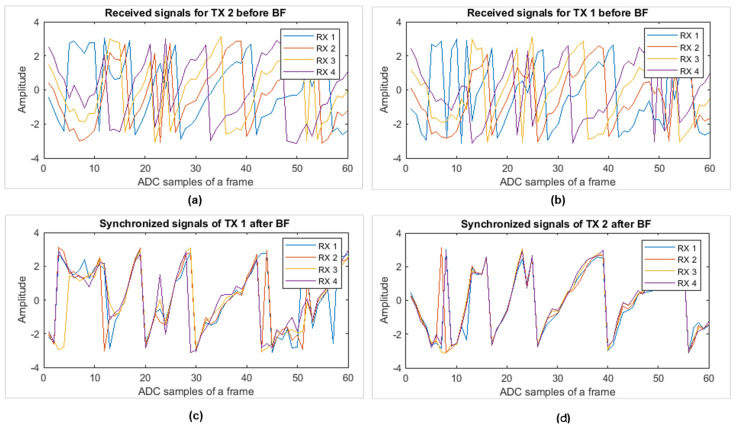
Phase of signal at target point: (**a**) raw data of all four RX channels corresponding to TX 1; (**b**) all RX corresponding to TX 2; (**c**) beamforming output corresponding to TX 1; and (**d**) the beamforming output corresponding to TX 2.

As stated in the earlier section, based on the intended target position, a delay (weight) vector was calculated, and a dot product was taken with the RDC as expressed in Equation (10). Afterwards, at the point where the target is located, we accumulated the phase of the signal before and after beamforming for each receiving channel, and the corresponding results are reported in Figure 10. The phases of the received raw signal for each TX channel before performing beamforming are not aligned with each other as expressed in Figure 10a,b. Figure 10a shows each received signal corresponding to TX 1. Similarly, Figure 10b shows the ADC samples of each RX channel corresponding to TX2. All these RX signals are multiplied with the weight vectors calculated based on Equation (8) and added together. The corresponding synchronized signals for TX 1 and 2 are shown in Figure 10b,c respectively. Note that in Figure 10, the phase adjustment is visualized against the raw data collected at each receiving channel.

### 4.5. Error Analysis with and without the Beamforming Case

The values of heart rate and the breathing rate extracted with radar were compared with the reference sensor. The difference between the radar and the reference sensor values was computed in terms of Mean Absolute Error (MAE) as:

(11) $$\mathrm{MAE}=\sum \frac{\left|VS_{i\_radar}-VS_{i\_reference}\right|}{n}\;,$$

where $VS_{radar}$ and $VS_{reference}$ denote the vital signs extracted with the radar and the reference sensor, respectively, and n represents the total number of observations for a particular instance. The MAE is widely used while investigating the level of agreement between radar and reference sensors for vital sign extraction [33,34]. In addition to that, in order to quantify the improvement in terms of percentage we used the below formula

(12) $$Improvement\;\left(\%\right)=\frac{{\mathrm{MAE}}_{BF}-{\mathrm{MAE}}_{without\;BF}}{{\mathrm{MAE}}_{without\;BF}}\left(100\right),\;$$

where ${\mathrm{MAE}}_{BF}$ and ${\mathrm{MAE}}_{without\;BF}$ represent the Mean absolute error with and without beamforming cases, respectively.

#### 4.5.1. Error Analysis for BR Extraction

The preliminary results show that the breathing rate does not improve noticeably while using beamforming. Perhaps a degradation was seen in the breathing rate extraction at a few locations. The rest of the details regarding the MAE comparison for the case of BR extraction are reported in Table 4. Based on the observation, the MAE for BR was already lower (in comparison to HR). For breathing rate extraction, the data from a single RX can be used to extract human vital signs.

#### 4.5.2. Error Analysis for HR Extraction

In contrast to breathing rate, heart rate showed better results when beamforming was applied. Since we intend to use beamforming mainly for HR extraction, the MAE for each distance and angle are discussed in detail in this section. The MAE analysis at each distance revealed that with a range of 0.9 m the radar was able to extract vital signs uniformly at all the angles. It can be said that at 0.9 m, the performance of the considered OTS radar is the same at different angles. As a result, negligible or no improvement was observed as expressed in Table 4 which shows the HR MAE comparison with and without beamforming cases at 0.9 meters’ distance. One of the possible reasons could be the presence of side lobes in the close proximity of the radar. A negative percentage of improvement in Table 5 corresponds to the case when the MAE for beamforming was higher than without beamforming. Although the (negative) improvement value is high in Table 5, the actual MAE with and without beamforming does not have a considerable difference at a distance of 0.9 m

Similarly, Table 6 shows the MAE for each angle at a distance of 1.2 m. It can be observed from Table 6 that except zero AoA, the percentage of improvement is always positive at all angles except at zero degrees. In comparison to the case of 0.9 m distance, the individual MAE values at 1.2 m shows higher improvement when beamforming was adopted. At zero degrees, a slight degradation was observed for HR extraction. Without beamforming, the MAE at zero degrees was 3.13, whereas with beamforming, the error was slightly increased to 3.52. In addition, as we move away from zero degree AoA, the improvement increased.

Table 7, shows the MAE for the 1.5 meters’ case. where an increase in the improvement in terms of percentage can be observed at most of the angles. At an extreme angle of 40 degrees, 29% improvement was observed which is the highest value of improvement observed in our experiments.

The comparison with and without beamforming at each angle and all the distances is shown in Table 8. It can be seen that except for zero degrees, beamforming always improved the overall performance of vital sign extraction. At an extreme angle of 40 degrees, without adopting beamforming, the error was 3.40 beats per minute, whereas with beamforming the error was 2.83 breaths per minute, resulting in an overall improvement of 16.54 %. At −40 degrees, the error reduced from 3.27 to 2.92 beats per minute which accounts for a 10.74 % improvement. The rest of the details of heart rate extraction are shown in Table 8.

As discussed earlier, beamforming at 0.9 m does not contribute significantly to reduce the MAE. Table 9 presents the results of the MAE with and without beamforming at 1.2 and 1.5 m only. The overall trend in Table 9 shows that with the exception of zero degrees, the MAE is reduced while adopting beamforming.

Based on the results shown in Table 4, Table 5, Table 6, Table 7, Table 8 and Table 9, we can conclude that for heart rate extraction, beamforming can be adopted, whereas for the breathing rate, radar without beamforming can be used to improve the performance of vital sign measurement.

**Figure 11:**
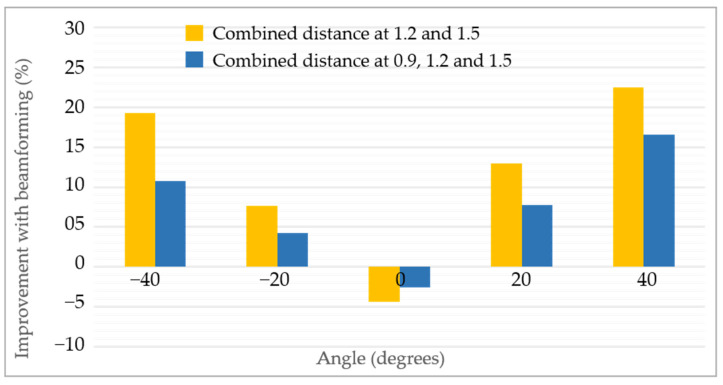
Improvement quantification of heart rate extraction when beamforming was applied.

As mentioned earlier, the MAE for the HR reduced when adopting beamforming; the summary of improvement is shown in Figure 11. The horizontal axis in Figure 11 shows each angle under-consideration, and the vertical angle shows the percentage of improvement with and without beamforming. The blue bar in Figure 11 shows the percentage of improvement at each angle when all three distances, which are 0.9, 1.2 and 1.5 m, were considered, whereas the yellow bar shows the percentage of improvement at 1.2 m and 1.5 m. It can be seen that at zero degree AoA, a negative improvement is seen which means that when the target is located in the middle, beamforming does not reduce the error between radar and reference sensor. On the other hand, as we start moving towards the extremes, the improvement becomes more prominent and visible. The overall trend in both the cases is consistent as seen in Figure 11.

## 5. Discussion

In order to utilize beamforming optimally for vital sign extraction, it should be noted that heart rate extraction is greatly improved with beamforming. Breathing rate, on the other hand, does not improves at all distances and angles. In addition, the MAE of the heart rate at zero degrees was increased; hence, at zero degree AoA without beamforming, data can be used. If the arrival horizontal angle is 20 degree or greater, beamforming improves heart rate extraction. If the human target is placed near the radar, the radar measures the vital signs equally at different angles. Note that we used the desired angle based on the ground-truth information while performing the beamforming

By accumulating the phase of the range-point where the human is located, we can confirm whether the applied delays have synchronized the signal properly or not. For reference, the signal before and after beamforming in Figure 10a–d can be observed.

## 6. Conclusions and Further Work

This paper presents the experimental comparison of vital sign extraction with and without beamforming at various angles and distances. The data were collected simultaneously for both the cases and the accuracy at each point was reported. Four different angles (zero, ±20, ±40) and three different distances (0.9, 1.2 and 1.5 m) were considered. Preliminary results show that the MAE of HR extraction reduces significantly at extreme angles. In addition, at a distance of 0.9 m, the effectiveness of beamforming was lowest compared to the other two distances (1.2 and 1.5 m). This research work presents a simple yet effective beamforming approach for vital sign extraction. It can be concluded that a conventional beamforming approach can be used to improve the vital sign extraction at the side angles. In future, we aim to consider complex and adaptive beamforming approaches such as the Minimum Variance Distortion-less Response (MVDR) beamforming method.

## Figures and Tables

**Table 1:** Parameters of OTS radar used in this study.

Parameters	Value
Number of TX antennas ($N_{TX}$)	2
Number of RX Antennas ($N_{RX}$)	4
Virtual receiving channels (VULA)	8
Starting Frequency ($f_{c}$)	60 GHz
Bandwidth (B)	3.89 GHz
Chirps in one frame	50
Frame Rate (per second)	20
Range Resolution ($d_{res}$)	4 cm
Maximum Range ($d_{max}$)	11 m

**Table 2:** Details of participants involved in this research work.

Participant	Age (Years)	Height (Centimeters)	Weight (Kilograms)
Participant 1	27	173	71
Participant 2	31	176	83
Participant 3	32	165	68
Participant 4	30	174	69
Participant 5	25	172	67
Participant 6	28	177	78

**Table 3:** Difference between the actual (desired) angle and the angle extracted using the beam scanner shown in Figure 7.

Desired AoA	Calculated AoA	Difference
−40	−47	7
−20	−22	2
0	0	0
20	23	3
40	47	7

**Table 4:** The MAE of BR at five different angles. Individual MAE at all three distances (0.9, 1.2 and 1.5 m) are combined at each angle.

Angle	Without Beamforming	With Beamforming	Improvement (%)
−40	1.77	1.51	14.42
−20	1.53	2.03	−32.78
0	1.16	1.04	10.74
20	1.44	1.15	20.29
40	1.25	1.51	−21.67

**Table 5:** The MAE of HR at 0.9 m.

Angle	Without Beamforming	With Beamforming	Improvement (%)
−40	2.47	2.84	−15.28
−20	2.91	3.06	−5.16
0	3.14	3.52	−11.91
20	3.40	3.46	−1.73
40	2.80	2.78	0.72

**Table 6:** The MAE of HR at 1.2 m.

Angle	Without Beamforming	With Beamforming	Improvement (%)
−40	4.11	3.27	20.25
−20	4.18	3.52	15.79
0	3.13	3.24	−3.50
20	3.95	3.74	5.37
40	2.75	2.46	10.51

**Table 7:** The MAE of HR at 1.5 m.

Angle	Without Beamforming	With Beamforming	Improvement (%)
−40	3.24	2.64	18.51
−20	3.13	3.20	−2.33
0	3.93	3.71	5.59
20	3.13	2.47	20.88
40	4.64	3.26	29.65

**Table 8:** The MAE of HR at all three distances (0.9, 1.2 and 1.5 m).

Angle	Without Beamforming	With Beamforming	Improvement (%)
−40	3.27	2.92	10.74
−20	3.41	3.26	4.28
0	3.40	3.49	−2.59
20	3.49	3.22	7.70
40	3.40	2.83	16.54

**Table 9:** The MAE of HR at five different angles. Individual MAE at all three distances (0.9, 1.2 and 1.5 m) are combined at each angle.

Angle	Without Beamforming	With Beamforming	Improvement (%)
−40	3.68	2.96	19.31
−20	3.66	3.36	7.63
0	3.53	3.48	−4.42
20	3.54	3.10	12.93
40	3.70	2.86	22.53
